# A survey of knowledge and use of telehealth among veterinarians

**DOI:** 10.1186/s12917-019-2219-8

**Published:** 2019-12-30

**Authors:** Kylie Watson, Julia Wells, Manoj Sharma, Stanley Robertson, John Dascanio, Jason W. Johnson, Robert E. Davis, Vinayak K. Nahar

**Affiliations:** 1 Center for Animal and Human Health in Appalachia, College of Veterinary Medicine, Lincoln Memorial University, Harrogate, TN USA; 2 Department of Behavioral & Environmental Health, School of Public Health, Jackson State University, Jackson, MS USA; 3 School of Health Sciences, Walden University, Minneapolis, MN USA; 4 Health for All, Omaha, NE USA; 5 Texas Tech School of Veterinary Medicine, Amarillo, TX USA; 6 Substance Use and Mental Health Laboratory, Department of Health, Human Performance and Recreation, University of Arkansas, Fayetteville, AR USA; 7 Department of Dermatology, School of Medicine, University of Mississippi Medical Center, 2500 North State Street – L216, Jackson, MS USA; 8 Department of Preventive Medicine, School of Medicine/John D. Bower School of Population Health, University of Mississippi Medical Center, Jackson, MS USA

**Keywords:** Telehealth, Telemedicine, Awareness, Practice, Education, Policy

## Abstract

**Background:**

As usage of digital information and communication technologies continues to grow, the incorporation of telehealth and telemedicine has become a topic of interest in the veterinary industry. Veterinary telemedicine presents the opportunity to expand veterinary medicine by increasing access to healthcare services for clients and patients and improving medical quality. The objective of this study was to assess veterinarians’ knowledge and utilization of telehealth and telemedicine.

**Results:**

Seventy-six veterinarians participated in the study and both qualitative and quantitative analyses were performed on the data collected. Several key themes emerged from the qualitative analysis of open-ended questions, including *telecommunication*, *Doctor of Veterinary Medicine (DVM)-patient services*, and *remote interaction*, among others. Through coding and qualitative analysis, researchers identified a lack of knowledge of the American Veterinary Medical Association (AVMA) definitions of telehealth and telemedicine. Specifically, a notable amount of participants were unaware of the distinction between the two practices per AVMA guidelines. Quantitative analyses revealed that the largest group of respondents reported sometimes utilizing telehealth and telemedicine in practice, with no distinct difference in utilization among the different age demographics of participants.

**Conclusions:**

These observations indicate a need for interventions both in veterinary school and continuing education programs with the purpose of increasing both knowledge and utilization of telehealth and telemedicine among veterinarians. While these recommendations serve as a starting point, future studies are needed to further enhance the understanding of veterinary telehealth and telemedicine in practice.

## Background

The use of telehealth within veterinary medicine has been an emerging topic of interest, particularly with the advancement of digital information and communication technologies (ICTs) [1]. According to the American Veterinary Medical Association (AVMA), veterinary telehealth is defined as an overarching term that describes all uses of technology to deliver healthcare information, education, and services remotely [2]. The AVMA breaks down the umbrella term telehealth into subcategories: telemedicine, teletriage, tele-advice, teleconsulting, telecommunication, telesupervision, telemonitoring, e-VFD, and e-prescription [2]. Based on the AVMA definitions, telemedicine is a subcategory of telehealth that involves the digital exchange of information from a distance regarding a patient’s clinical health status within an existing Veterinarian-Client-Patient-Relationship (VCPR) [2, 3]. Though the AVMA distinguishes between the two, telehealth and telemedicine are often used interchangeably in literature and clinical settings. It is apparent that there is not a broad consensus or strict definition. For the purpose of this study and discussion, the definitions of telehealth and telemedicine provided by the AVMA were used.

Veterinary telehealth is not a new concept, and many veterinarians likely practice without even appreciating that the use of basic communication platforms, including a telephone, fax, or email to discuss a case, constitutes telehealth [4]. In the 1980s, the first use of a transtelephonic electrocardiogram transmitter connected distant veterinarians with specialized cardiologists in New York [4, 5]. The cardiologists could then collaborate with the referring veterinarian on a probable diagnosis, prognosis, and therapeutic recommendations [4], an example of teleconsulting, a subcategory of telehealth [2]. However, veterinary telehealth likely appeared years prior, back to the early days of the telephone or telegraph [5]. In more recent times with advancements in technology and faster networks, telehealth has assumed newer dimensions and now includes a broad range of available platforms.

The rise in development of new digital ICTs has presented more opportunities to expand the practice of veterinary medicine by incorporating the tools of telehealth. Videoconferencing as a means to conduct examinations, instead of an in-clinic visit, is a telehealth opportunity of interest. In a study focusing on videoconferencing for postsurgical recheck appointments, researchers found that owners in the telemedicine group were satisfied with a virtual appointment and also reported that their dogs were less afraid compared with what was typical for them during an in-clinic appointment [6]. Video conference appointments, in addition to other means of veterinary telehealth, could provide alternatives to healthcare visits for high anxiety or stressed patients and should be further explored. Temperature, pulse rate, respiratory rate, and blood pressure are the main vital signs that are often measured during a veterinary physical examination, and are parameters that can be affected by outside factors, such as stress [7]. When comparing those four major vital signs in healthy dogs in a home environment and in a veterinary hospital, researchers found that there were significant differences in blood pressure, rectal temperature, and pulse rate [7]. As variations in vital signs can affect diagnostic capabilities [7], telehealth should be considered as a viable option for assessment of patients.

Wearable biosensors for animal health management is another application of telehealth, providing the opportunity to improve medical quality and access to healthcare. These wearable technologies, if built precisely and used correctly, can monitor body temperature, detect stress, observe behavior and movement, and detect the presence of viruses and pathogens, among other parameters [8]. Smartphone apps tracking these vitals and behaviors allow veterinarians access to the herd remotely and able to provide faster and more accurate medical assistance [8].

With the rapid evolution of digital technologies, veterinary providers must adopt telehealth services to stay relevant and better serve patients and their owners. While obtaining the equipment and programs to offer these telehealth services is important, assuring veterinarians’ understanding of telehealth, its benefits, and ultimately their practice thereof is vital to its success. Therefore, the objective of this current study was to assess veterinarians’ knowledge and utilization of telehealth and telemedicine. The information gained from this study will be beneficial to the design and implementation of future intervention programs to improve telehealth practice among veterinarians.

## Results

Table 1 displays socio-demographic characteristics of the participants. A total of 76 participants responded to the survey, which was sent to 282 veterinarians. The largest age group category reported was 41–50 years old (25.0%). The majority of the participants were males (60.5%) and 92.1% of the participants identified themselves as White/Caucasians. Approximately 40% of the participants reported working at the clinics that averaged $1,000,000 - $5,000,000 gross income annually.

**Table 1:** Socio-demographic Information of the Participants (*N* = 76)

	*n* (%)
Age	
20–30 years	1 (1.3)
31–40 years	18 (23.7)
41–50 years	19 (25.0)
51–60 years	16 (21.1)
Over 60 years	19 (25.0)
Gender	
Male	46 (60.5)
Female	27 (35.5)
Race/ethnicity	
White or Caucasian American	70 (92.1)
Black or African American	1 (1.3)
Hispanic American	2 (2.6)
Annual Gross Revenue of Practice	
Less than $250,000	1 (1.3)
$250,000 to $500,000	3 (3.9)
$500,001 to $750,000	4 (5.3)
$750,001 to $1,000,000	5 (6.6)
$1,000,000 to $2,000,000	22 (28.9)
$2,000,001 to $5,000,000	16 (21.1)
More than $5,000,000	13 (17.1)
Prefer Not to Answer	7 (9.2)

*Due to missing data, the percentage of responses in each category of variables do not sum to 100%*

Regarding practice information, practices employed an average of 6.85 veterinarians. The majority of the practices were private (60.5%) and either companion animal exclusive (57.9%) or companion animal predominant (15.8%). Suburban practices were the most common (55.3%) followed by rural (31.6%) and urban (6.6%). Most participants identified themselves as practice owners (60.5%) or associate veterinarians (14.5%).

The four open-ended questions were analyzed using open-ended coding and thematic analysis. Individual responses were often sorted into more than one theme; therefore, the percentages of themes for each question do not sum to 100%. Question 10, “How would you define telehealth?” had a response rate of 81.6%. Five themes emerged based on the provided responses: *telecommunication* (55.3%), *remote interaction* (35.5%), *Doctor of Veterinary Medicine* (*DVM)-DVM interaction* (13.2%), *DVM-client interaction* (29.0%), and *DVM-patient services* (46.1%). *Telecommunication*, the most common theme for question 10, included any responses mentioning technology platforms such as video, phone, text, email, facetime, social media, or the Internet. *Remote interaction* included responses that referred to distance, a lack of hands-on medicine or physical exam, or nontraditional, remote, or indirect contact. Responses referencing communication between veterinarians, including specialty consultation and collaboration, constituted *DVM-DVM interaction*, while *DVM-client interaction* included responses mentioning an exchange between a veterinarian and a client. The second most common theme in question 10, *DVM-patient services*, included responses that mentioned medical services performed by a veterinarian such as patient healthcare, treatment, diagnosis, and medication prescription.

Question 12, “How would you define telemedicine?” had a response rate of 71.1%. Five of the seven themes developed were defined identically to those used in question 10: *telecommunication* (31.6%), *remote interaction* (25.0%), *DVM-DVM interaction* (4.0%), *DVM-client interaction* (15.8%), and *DVM-patient services* (34.2%). The additional theme *accessibility/improvements to healthcare* (10.5%) pertained to any responses mentioning the ease of access to medical care, timeliness, and enhancement of healthcare. The final theme from question 12, *same as telehealth* (11.8%), simply pertained to responses in which the respondent believed telehealth and telemedicine to be identical. The category of *DVM-patient services* was the most common theme found in question 12, followed by *telecommunication*.

Question 11, “What keywords come to mind when you think of telehealth?” had a response rate of 80.3%. Eight themes developed based on the responses provided: *financial related* (9.2%), *telecommunication* (32.9%), *DVM services/diagnostics* (18.4%), *remote interaction* (15.8%), *accessibility to healthcare* (18.4%), *legality and VCPR* (9.2%), *concern and uncertainty* (13.2%), and *improving veterinary medicine* (13.2%). The *financial related* category included responses that mentioned monetary aspects such as revenue or income. It was further divided into sub-themes: *gain of income/revenue (5.3%)* and *loss of income/revenue (4.0%)*. Responses grouped into the themes *telecommunication* and *remote interaction* were classified based on previous theme definitions. *DVM services/diagnostics* referred to any responses that mentioned diagnostic procedures or veterinary consultation. Responses that referenced ease of access to healthcare, convenience, or increased availability were included in the theme of *accessibility to healthcare*. The theme *legality and VCPR* incorporated responses that mentioned legality, liability, or the veterinary client-patient relationship (VCPR). Responses that portrayed hesitation or negativity toward telehealth were classified into *concern and uncertainty*. Conversely, *improving veterinary medicine* included responses that portrayed telehealth as an advancement to healthcare and the opportunity for collaboration.

Question 13, “What keywords come to mind when you think of telemedicine?” had a response rate of 63.1%. Eight of the nine themes developed were defined identically to those used in question 11: *financial related* (5.3%), *telecommunication* (22.4%), *DVM services/diagnostics* (15.8%), *remote interaction* (11.8%), *accessibility to healthcare* (11.8%), *legality and VCPR* (9.2%), *concern and uncertainty* (9.2%), and *improving veterinary medicine* (11.8%). The additional theme from question 13, *same as telehealth* (7.9%), simply pertained to responses indicating that the respondent believed telehealth and telemedicine to be identical. *Telecommunication* was the most common theme found in question 13, followed by *DVM services/diagnostics*.

Table 2 displays responses to questions 14 and 15, which asked respondents if they utilize veterinary telehealth or telemedicine, respectively, in practice. Regarding the utilization of telehealth, the majority of respondents sometimes utilize telehealth (21.1%), followed by never utilize telehealth (19.7%). Utilization of telemedicine followed the same trend, with 22.4% of respondents reporting sometimes utilizing telemedicine and 19.7% never utilizing telemedicine.

**Table 2:** Utilization of Telehealth and Telemedicine in Practice (*N* = 76)

	*n* (%)
Utilization of Telehealth	
Never	15 (19.7)
Hardly Ever	12 (15.8)
Sometimes	16 (21.1)
Fairly Often	10 (13.2)
Often	4 (5.3)
Utilization of Telemedicine	
Never	15 (19.7)
Hardly Ever	11 (14.5)
Sometimes	17 (22.4)
Fairly Often	11 (14.5)
Often	2 (2.6)

*Due to missing data, the percentage of responses in each category of variables do not sum to 100%*

Table 3 details different platforms veterinarians used for various interactions with clients and patients. The office visit was the most commonly selected platform for all interactions with except for after-hours calls/emergencies. The phone was the second most commonly selected platform in all categories except for after-hours calls/emergencies, in which it was most common (39.5%), and client education, in which email was the second most commonly selected platform (48.7%). As a follow up to question 16, participants estimated what percentage they utilized each platform for a client and patient interactions. While exact percent utilization varied among respondents, the office visit was the most frequently utilized platform overall based on the percentages given.

**Table 3:** Platform Utilization for Interaction with Clients and Patients (*N* = 76)

	Office Visit *n* (%)	Phone *n* (%)	Text *n* (%)	Email *n* (%)	(1-way) *n* (%)	(2-way) *n* (%)
Initial visit/consultation	48 (63.2)	20 (26.3)	13 (17.1)	16 (21.1)	4 (5.3)	0 (0)
Follow-up	45 (59.2)	39 (51.3)	22 (28.9)	27 (35.5)	4 (5.3)	2 (2.6)
Triage	43 (56.6)	25 (32.9)	11 (14.5)	12 (15.8)	4 (5.3)	1 (1.3)
After hours calls/emergencies	25 (32.9)	30 (39.5)	13 (17.1)	6 (7.9)	4 (5.3)	2 (2.6)
Pre-surgery visit	45 (59.2)	18 (23.7)	10 (13.2)	12 (15.8)	1 (1.3)	1 (1.3)
Post-surgery visit	45 (59.2)	31 (40.8)	16 (21.1)	17 (22.4)	3 (3.9)	1 (1.3)
Client Education	45 (59.2)	36 (47.4)	22 (28.9)	37 (48.7)	6 (7.9)	3 (3.9)

*Due to missing data, the percentage of responses in each category of variables do not sum to 100%*

Table 4 presents responses from question 18, in which respondents selected platforms specifically used to advise clients. In-person was the most frequently selected platform for general advice to an established client (61.8%), medical advice to an established client with a new patient (60.5%), and medical advice to a non-client (42.1%). The phone was the most frequently selected platform for general advice to a non-client (44.7%) and medical advice to an established client with a previously seen patient (60.5%).

**Table 4:** Platform Utilization for Client Advisement (*N* = 76)

	In Person *n* (%)	Phone *n* (%)	Text *n* (%)	Email *n* (%)	(1-way) *n* (%)	(2-way) *n* (%)
General advice–established client	47 (61.8)	46 (60.5)	24 (31.6)	30 (39.5)	2 (2.6)	1 (1.3)
General advice–non-client	33 (43.4)	34 (44.7)	6 (7.9)	16 (21.1)	1 (1.3)	1 (1.3)
Medical advice–established client with previously seen patient	45 (59.2)	46 (60.5)	23 (30.3)	31 (40.8)	3 (3.9)	3 (3.9)
Medical advice–established client with new patient	46 (60.5)	29 (38.2)	8 (10.5)	16 (21.1)	1 (1.3)	1 (1.3)
Medical advice–non-client	32 (42.1)	19 (25.0)	3 (3.9)	5 (6.6)	1 (1.3)	1 (1.3)

*Due to missing data, the percentage of participants in each category of variables do not sum to 100%*

For question 19, “Who would you consider an established client,” the majority of respondents (42.1%) considered established clients, someone, they have seen within the past year. The remaining options - someone that has been seen within 6 months, 1.5 years, 2 years, or 3 years, comprised the rest of the responses. Considering an established client as someone that has been seen within the past 6 months was the second most common response, accounting for 9.2% of respondents.

## Discussion

The objective of this study was to assess veterinarians’ knowledge and utilization of telehealth and telemedicine. Data was collected via a 19-question survey that was sent to veterinarians affiliated with a private southern college of veterinary medicine. These veterinarians were selected because of their association with the college’s distributed clinical year program and have had students who have rotated through their clinical practices to obtain clinical experience. Veterinarians that participated in this study were from various areas of the United States, but were primarily from the southern and eastern United States. By conducting this study, researchers hope to identify targets for interventions related to the understanding and utilization of telehealth and telemedicine among veterinarians. As ICTs continue to develop, more opportunities arise to expand the practice of veterinary medicine. Veterinary telehealth offers an opportunity to change the face of veterinary medicine, improving the lives of veterinarians, clients, and patients.

The AVMA defines telehealth as an umbrella term that encompasses all usage of technology to deliver health care information, education, or care remotely, while telemedicine, a subset of telehealth, refers specifically to healthcare services provided to improve a patient’s health status and requires the existence of a VCPR [2, 3]. Interestingly, when asked the definition of telemedicine, 9 of 54 respondents for the question responded “same” or “same as telehealth.” Further, additional responses to the definition of telemedicine by some participants were identical to those they gave for the definition of telehealth. This demonstrates a lack of knowledge of the AVMA definitions of telehealth and telemedicine in veterinary practice.

As previously stated, a broad consensus seems lacking in the veterinary community on the distinction between telehealth and telemedicine; however, the AVMA published definitions of the terms in an attempt to clarify discrepancies among telehealth and its subsets. One of the most important differences between telehealth and telemedicine, pere the AVMA published definitions, is that telemedicine requires the existence of a VCPR, a relationship essential to proper treatment [2, 3]. Qualitative data reflected the importance of the VCPR leading to the establishment of *legality and VCPR* as a theme. A current concern for the use of telemedicine is if a valid VCPR can be established remotely, or if it must be established through an in-person clinic/site visit. The AVMA holds the stance that a physical exam or site visit is required to form a VCPR [9]. Under this stance, telemedicine cannot be used for an initial consultation with new patients; veterinarians must physically observe or examine all new patients before advising clients and diagnosing or treating patients [9]. Contrary, to the AVMA guidelines, the American Association of Veterinary State Boards (AAVSB) proposed a model in 2018 where it is possible to establish a VCPR remotely [10]. States can now use their discretion as to which guidelines to follow regarding the proper establishment of a VCPR [10]. Some states, suh as California, have adopted policy that explicitly forbids the remote establishment of a VCPR [11]; whereas other states, such as Oklahoma, allow for the remote establishment of a VCPR in certain cases [12]. Similar conflict regarding telemedicine among governing medical bodies has surfaced in human medicine. The American Medical Association states that a patient-physician relationship must be established prior to providing telemedicine services [13], while the Federation of State Medical Boards abides by a policy that a patient-physician relationship may be established using telemedicine provided that the standard of care is still met [14].

In addition to knowledge, utilization of telehealth and telemedicine was also assessed. Almost half of the respondents, regardless of age demographic, selected never or hardly ever utilizing telehealth or telemedicine. The effects of age on telemedicine utilization have been investigated previously with conflicting results [15, 16]. While one study found that veterinarians over the age of 60 endorsed electric communication more frequently than their younger counterparts [15], another found that younger age groups of owners, veterinarians, and students held a more positive attitude toward telemedicine than the older age group [16]. Of the survey responsdents who reported using telehealth, 1-way and 2-way video were the least reported telecommunication platforms utilized. These observations indicate a need for interventions both in veterinary school and continuing education programs to educate veterinary students and veterinarians on the benefits of utilizing telehealth in practice. One study in human medicine found that practitioners who have utilized telehealth previously were more likely to use telehealth in the future than those who have not [17]. Early interventions in veterinary school and practices could promote the concept of telehealth to non-adopters and possibly increase the chances that they would consider utilizing telehealth in the future. Specific interventions could explore how to increase video telecommunication usage in veterinary clinics, as the video may be the best alternative to a hands-on physical exam. One telemedicine study found that participants who utilized videoconferencing for appointments had an overall high satisfaction rate with the platform and preferred use of videoconference to in-person clinic visits [6].

Responses to open-ended questions about keywords related to telehealth and telemedicine manifested into multiple themes, one of which was *concern and uncertainty*. These responses indicated that some felt negatively toward telehealth and thought that it could have drawbacks in practice. Negative feelings toward telemedicine have been investigated previously, with one study finding that only 20% of participants believed clients would willingly pay for telemedicine services [15]. One potential drawback for the utilization of telehealth in practice is that it could open the door for clients to expect veterinary consults at their convenience, any time of day. This expectation could have drastic effects on a veterinarian’s work-life balance and mental health. Furthermore, the utilization of telehealth in practice could cause a blurred line between free medical advice and paid remote consultations. For example, a veterinary clinic that offers telephone or video consultations to clients for a fee may be conflicted about charging clients a fee when answering questions as a follow up to in-person appointments. These potential discrepancies could create confusion in the clinic and therefore, clear regulations and guidelines must be established within the hospital and amongst clients to prevent such problems.

Another theme found in open-ended responses was *improving veterinary medicine*. These responses referred to telehealth and telemedicine as positive contributions to veterinary practice, with potential to expand veterinary healthcare. One important benefit of utilizing telehealth in practice is that it may enable veterinarians to better serve communities suffering from veterinary shortages by allowing for remote veterinary services. As of 2019, all except for six states in the United States have a designated veterinary shortage [18]. The utilization of telehealth can address these shortages without having a physical veterinary clinic operating in the area. For example, food animal and herd health operations could utilize telehealth by equipping animals with health monitoring sensors and transmit this information to a consulting veterinarian [8, 19]. Regarding small animal practices, the utilization of telehealth could benefit animals that experience stress during travel or in a clinic environment [6, 7].

In evaluating the results of this study, there are a few limitations to consider. The study sample size was relatively low at 76 participants. Additionally, the majority of respondents identified as male, which is not reflective of the veterinary industry overall [20]. Finally, a decrease in response rate was observed as respondents progressed through the survey, which caused some difficulty in comparing data among individual questions. Overall, the low response rate and demographic characteristics of respondents may severely limit generalizability of the results.

## Conclusion

Given the increase in digital ICTs, it is important that veterinarians understand telehealth and how to effectively utilize telehealth services in practice. Overall, researchers identified a lack of both knowledge of the telehealth and telemedicine and its utilization among veterinarians surveyed. These findings stress the need for interventions both in veterinary school and continuing education programs to increase awareness of telehealth and its potential benefits in veterinary practice. The incorporation of telehealth into veterinary practice allows for the advancement of medicine, benefitting veterinarians, clients, and patients alike.

## Methods

### Participants and procedure

All veterinarians affiliated with a private southern college of veterinary medicine were recruited to participate in this cross-sectional research study. Veterinarians were contacted via e-mail, informing them of the research study, its purpose, and a link to the online questionnaire. As clearly noted in the e-mail, upon clicking the survey link, the veterinarians were giving their electronic consent to participate in the study. All responses to the questionnaire were collected anonymously and kept confidential. Data were collected between 2/19/2019–4/14/2019, and a total of four reminder e-mails were sent to all recruited participants. Participation was voluntary and participants could withdraw at any time during the period of this study. The study research was reviewed and approved by the university Institutional Review Board (IRB; Protocol #759 V.0).

### Instrumentation

The instrument (Additional file 1: Telehealth Practice Survey) was developed by researchers with previous experience in survey-based studies. Due to the exploratory nature of the study, open-ended questions were developed. All of the questions were discussed among the researchers prior to finalizing the questionnaire. Once developed, the survey was sent to two veterinarians to establish face and content validity.

Upon clicking the link and being directed to the 19-question online survey, participants’ knowledge and utilization of veterinary telehealth and telemedicine were assessed. The survey was estimated to take approximately five minutes to complete. Questions 1–3 inquired about basic socio-demographic participant information, including gender, age, and race/ethnicity. Questions 4–9 inquired about the type of practice each veterinarian was engaged in, role in practice, number of full-time veterinarians in their practice, species generally seen, annual gross revenue of the practice, and geographic location.

In open-ended responses, the participants defined telehealth in question 10 and telemedicine in question 12. In question 11 and 13, participants listed keywords that came to mind when thinking of telehealth and telemedicine. The degree to which veterinary telehealth and telemedicine were utilized in practice by the participants was assessed in questions 14 and 15. Question 16 inquired about the ways that participants interacted with clients and patients. Participants were then asked to estimate a percentage for each platform utilized in question 17. In question 18, participants selected various ways they give advice to clients. Lastly, question 19 assessed how participating veterinarians defined an established client.

### Data analyses

For qualitative data, a thematic analysis [21] based on Bernard and Ryan’s (1998) approach was performed in a two-step process. In the first step, two researchers independently analyzed responses to the open-ended questions and identified sets of common themes found among the responses provided. In the next step, using those themes previously identified, researchers independently coded the responses. Researchers then convened to discuss discrepancies and a common set of themes were agreed upon. Frequencies of common themes were calculated into percentages. Quantitative data analyses were performed using IBM SPSS statistical software version 25.0.

## Supplementary information

**Additional file 1.** Telehealth Practice Survey. Survey instrument to assess information about veterinarians’ knowledge and utilization of telehealth.

## Data Availability

The datasets used and/or analyzed during the current study are available from the corresponding author on reasonable request.

## Abbreviations

AAVSB: American Association of Veterinary State Boards
AVMA: American Veterinary Medical Association
DVM: Doctor of veterinary medicine
ICTs: Information and communication technologies
IRB: Institutional Review Board
VCPR: Veterinarian-client-patient-relationship
