# Supplementary material for: A survey of knowledge and use of telehealth among veterinarians
Source: BMC Vet Res. 2019 Dec 30;15:474. doi: 10.1186/s12917-019-2219-8 (PMC6937652; doi:10.1186/s12917-019-2219-8)
Supplement: Supplementary file 1 — Additional file 1. Telehealth Practice Survey. Survey instrument to assess information about veterinarians’ knowledge and utilization of telehealth. [file 12917_2019_2219_MOESM1_ESM.docx]

Telehealth Practice Survey

This survey is designed to help provide information about veterinarians' knowledge and utilization of telehealth. Your responses will help in developing effective veterinary telehealth programs for veterinarians and help to  improve the development of successful telehealth programs or policies. All data from this survey will be kept confidential.  If you are unsure about a question, please try to answer it to the best of your abilities. Thank you for your help!
Click the next arrow below to continue

Q1 **What is your gender?**

- Male
- Female
- Other _________________

Q2 **How old are you today?**

- 20-30 years
- 31-40 years
- 41-50 years
- 51-60 years
- Over 60 years

Q3 **What is your race/ethnicity?**

- White or Caucasian American
- Black or African American
- Asian American
- American Indian
- Hispanic American
- Other _________________

Q4 **What type of practice are you engaged in?**

- Private Practice – Owner
- Private Practice – Associate
- Corporate / Group Practice
- College or University
- Government – Federal
- Government – State or Local
- Industry
- Uniformed Service
- Other, not listed, please write _________________

Q5 **What best describes your role in the practice?**

- Owner
- Associate
- Other, not listed, please write _________________

Q6 **How many veterinarians (full-time equivalent) are in your practice? (please enter number)**

________________________________________________________________

Q7 **What species do you generally see?**

- Companion Animal Exclusive
- Companion Animal Predominately
- Equine
- Food Animal
- Mixed Animal
- Other _________________

Q8 **What is the annual gross revenue of your practice?**

- Less than $250,000
- $250,000 to $500,000
- $500,001 to $750,000
- $750,001 to $1,000,000
- $1,000,000 to $2,000,000
- $2,000,001 to $5,000,000
- More than $5,000,000
- Prefer not to answer

Q9 **What is the geographic location of your practice?**

- Urban
- Rural
- Suburban

Q10 **How would you define telehealth?**

Q11 **What keywords come to your mind when you think of telehealth?**

Q12 **How would you define telemedicine?**

Q13 **What keywords come to your mind when you think of telemedicine?**

Q14 **Do you utilize veterinary telehealth in your practice?**

- Never
- Hardly Ever
- Sometimes
- Fairly Often
- Often

Q15 **Do you utilize veterinary telemedicine in your practice?**

- Never
- Hardly Ever
- Sometimes
- Fairly
- Often

Q16 **Select the way(s) below that you interact with clients and patients:**

|  | Office Visit | Phone | Text | Email | Video  (1-way) | Video  (2-way) |
| --- | --- | --- | --- | --- | --- | --- |
| Initial visit/consultation |  |  |  |  |  |  |
| Follow-up |  |  |  |  |  |  |
| Triage |  |  |  |  |  |  |
| After hours calls |  |  |  |  |  |  |
| Pre-surgery visit |  |  |  |  |  |  |
| Post-surgery visit |  |  |  |  |  |  |
| Client education |  |  |  |  |  |  |
| Other |  |  |  |  |  |  |

Q17 **For the above question, what percentage do you utilize each of the available options.** **Example:  (total equals 100%)      90%      5%          5%         0%          0%       0%**

|  | Office Visit  (%) | Phone (%) | Text  (%) | Email (%) | Video  (1-way)  (%) | Video  (2-way)  (%) |
| --- | --- | --- | --- | --- | --- | --- |
| Initial visit/consultation |  |  |  |  |  |  |
| Follow-up |  |  |  |  |  |  |
| Triage |  |  |  |  |  |  |
| After hours calls/Emergencies |  |  |  |  |  |  |
| Pre-surgery visit |  |  |  |  |  |  |
| Post-surgery visit |  |  |  |  |  |  |
| Client education |  |  |  |  |  |  |
| Other |  |  |  |  |  |  |

Q18 **Select the way(s) below that you give advice to clients:**

|  | In Person | Phone | Text | Email | Video  (1-way) | Video  (2-way) |
| --- | --- | --- | --- | --- | --- | --- |
| General advice – established client |  |  |  |  |  |  |
| General advice – non-client |  |  |  |  |  |  |
| Medical advice – established client with previously seen patient |  |  |  |  |  |  |
| Medical advice – established client with new patient |  |  |  |  |  |  |
| Medical advice – non-client |  |  |  |  |  |  |
| Other |  |  |  |  |  |  |

Q19 **Who would you consider an established client?**

- Someone you saw within 6 months
- Someone you saw within 1 year
- Someone you saw within 2 years
- Someone you saw within 3 years
- Other (please specify) _________________
